# *relA* Enhances the Adherence of Enteropathogenic *Escherichia coli*

**DOI:** 10.1371/journal.pone.0091703

**Published:** 2014-03-18

**Authors:** Beny Spira, Gerson Moura Ferreira, Luiz Gustavo de Almeida

**Affiliations:** Departamento de Microbiologia, Instituto de Cêincias Biomédicas, Universidade de São Paulo, São Paulo-SP, Brazil; Institut National de la Recherche Agronomique, France

## Abstract

Enteropathogenic *Escherichia coli* (EPEC) is a known causative agent of diarrhea in children. In the process of colonization of the small intestine, EPEC synthesizes two types of adhesins, the bundle-forming pilus (BFP) and intimin. The BFP pilus is an adhesin associated with the initial stages of adherence of EPEC to epithelial cells, while the outer membrane protein intimin carries out the intimate adherence that takes place at the third stage of infection. BFP is encoded by the *bfp* operon located in plasmid EAF, present only in typical EPEC isolates, while *eae*, the gene that encodes intimin is situated in the LEE, a chromosomal pathogenicity island. Transcription of *bfp* and *eae* is regulated by the products of the *perABC* operon, also present in plasmid EAF. Here we show that deletion of *relA*, that encodes a guanosine penta and tetraphosphate synthetase impairs EPEC adherence to epithelial cells *in vitro*. In the absence of *relA*, the transcription of the regulatory operon *perABC* is reduced, resulting in lower levels of BFP and intimin. Bacterial adherence, BFP and intimin synthesis and *perABC* expression are restored upon complementation with the wild-type *relA* allele.

## Introduction

Enteropathogenic *Escherichia coli* (EPEC) is one of the causes of infant diarrhea in developing countries [1], [2]. Typical EPEC cells form microcolonies on epithelial cell monolayers, a pattern known as localized adherence (LA) [3]. These strains carry a large plasmid known as EAF, which harbors two operons, *bfp* and *perABC* (or *per*), needed to confer on EPEC the LA phenotype. The *bfp* operon is formed by 14 genes that are associated with the biogenesis of the bundle-forming pilus (BFP), a type IV fimbria found in typical EPEC strains [4]. *bfpA*, the first gene of the operon encodes the main subunit of the fimbria. BFP is required in the first stage of EPEC infection, i.e., for the formation of the bacterial clusters that will ultimately result in the LA pattern [5]–[7]. The second stage of EPEC infection is characterized by the secretion of bacterial effector via a type III secretion system into the host cell followed by the corruption of the host signal transduction. EPEC chromosome harbors a 36 kb pathogenicity island known as the locus of enterocyte effacement, LEE [8]. Many genes associated with EPEC virulence are present in the LEE, including the ones encoding the type III secretion system, the secreted proteins and the adhesin intimin. Intimin is implicated in the third stage of infection, which involves enterocyte effacement, pedestal formation at the enterocyte membrane and intimate bacterial attachment to the host cell [9], [10]. In typical EPEC, transcription of the *bfp* operon and of the LEE genes are activated, respectively, by PerA and PerC, encoded by the *perABC* operon [11], [12].

The nucleotides guanosine tetra and penta-phosphate, collectively referred as ppGpp, accumulate in response to adverse environmental conditions. It was first noticed in *E. coli* starved for amino acids [13] and later observed under other stress conditions, such as deprivation of carbon, nitrogen [14] and phosphate [15]. ppGpp accumulation is accompanied by the stringent response, a metabolic adjustment characterised by a dramatic decrease in stable RNA and ribosome synthesis, general protein inhibition and the synthesis of specific proteins, such as the alternative sigma factor RpoS [16], [17]. In *E. coli* ppGpp is synthesized by two related proteins - RelA and SpoT. The ribosome-bound RelA is activated by uncharged tRNAs under conditions of amino acid limitation or by the addition of inhibitors of aminoacyl tRNA synthases [18]. The bifunctional enzyme SpoT displays a strong hydrolase and a weak synthetase activity, owe to the presence of two functional domains [19]. SpoT senses several stress conditions, in a still unclear fashion, and usually responds by inhibiting its hydrolase activity [20]. Interestingly, though all wild-type strains respond in a similar fashion to nutrient limitation, the intrinsic levels of ppGpp are not constant throughout the *E. coli* species [21], [22].

Mutations in *relA* confer on the bacterium a relaxed phenotype, which consists of a continuous accumulation of stable RNA under amino acid starvation. This mutant, however, responds normally to other stress conditions. Artificial induction of ppGpp to high levels results in growth arrest and inhibition of protein synthesis [23]. ppGpp is associated with diverse cellular functions, such as rRNA synthesis, mRNA elongation, amino acids, carbohydrate and lipid metabolism, DNA replication and virulence [15], [24]. The mechanism through which ppGpp exerts so many pleiotropic effects is not entirely clear. It can be partially explained by the fact that ppGpp interacts directly with RNA polymerase and shifts the affinity of the core enzyme towards alternative sigma factors [25]. However, the effect of ppGpp on protein synthesis and other post-transcriptional events [17], [26] cannot be explained by its interaction with RNA polymerase.

Here we report the effect of *relA* and ppGpp on the synthesis of EPEC virulence factors. We show that deletion of *relA* impairs bacterial adherence, reduces the synthesis of the adhesins BFP and intimin and inhibits the transcription of the *perABC* operon.

## Materials and Methods

### Media and growth conditions

LB medium is as described [27]. T-salts medium is a Tris-buffered minimal medium supplemented with 0.2% glucose [28] and variable concentrations of KH_2_PO_4_. Amino-triazole (AT) plates were prepared as described [29]. Dulbecco's Modified Eagle's Medium (DMEM) is a medium for epithelial cells (Cultilab-Brazil). HEp-2 cells were cultured in flasks containing DMEM enriched with 10% fetal calf serum (FCS), 50 U penicillin and 50 μg/ml streptomycin at 37°C. The antibiotics were omitted in assays where bacteria were added. For overnight growth, bacteria were usually cultivated in LB medium, for all other purposes they were grown in DMEM. Growth rate was calculated according to the formula: 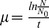, where N and N_0_ respectively correspond to initial and final OD_600_ of the exponential growth phase and t is time-course of the growth curve.

### Bacterial strains and plasmids

The strains and plasmids used in this study are described in Table 1.

**Table 1:** Bacterial strains, plasmids and DNA oligos used in this study.

Strains	Genotype	Source
BS230	BW25113 Δ*relA*::Cm	This study
BW25113	*E. coli* K-12 carrying the recombineering plasmid pKD46	[30]
CF1652	MG1655 *relA*::KmR	M. Cashel
CF12489	*argA*::Tn*10 relA* ^+^	M. Cashel
CP01	MG1655 *lacZ*::Tn*5*	lab collection
KM32	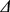 *recBCD*::P*tac-gam-bet-exo cat*	[54]
LRT9	EPEC O111:abH2	[33]
BS1230	LRT9 *bfpA*::SPA-Km	This study
BS1264	GMF204 *bfpA*::SPA-Km	This study
BS1298	LRT9 *eae*::SPA-Km	This study
BS1299	GMF204 *eae*::SPA-Km	This study
GMF204	LRT9 Δ*relA*::KmR	This study
GMF302	LRT9 *argA*::Tn*10 relA* ^+^	This study
LG01	LRT9 *lacZ*::Tn*5*	This study
**Plasmids**		
pACT3	Cloning vector	[55]
pBS32	pLG19 GmR	This study
pGM17	*per* promoter cloned upstream to the promoterless *lacZ*	[33]
pJL148	SPA tag source	[31]
pKNOCK-Gm	Plasmid carrying the gentamycin resistance (GmR) gene	[56]
pLG19	*relA* ^+^ cloned in plasmid pACT3 under P*tac*	This study
pTZ57R/T	Cloning vector	Thermo
**Oligos**		
bfpA813(F)-SPA	AAATACTGATTCAACCAATAAAGTTACATATTTTATGAAGTCCATGGAAAAGAGAAG	
bfpA813(F)-SPATR	CCCATATAATACGCCCAAAACAGGGCGTATTATGTAGATTCATATGAATATCCTCCTTAG	
eae-lrt9(F)-SPA	TGGAGTAAACAATAAGAATGCTTTTTCTGTTTGTGTAAAATCCATGGAAAAGAGAAGA	
eae-lrt9(R)-SPA	AATGAATTTTATTTTCCGGGATTTGAGATGTAATTAAATTCATATGAATATCCTCCTTAG	
relA1-fw-P1-fw	ATGGTTGCGGTAAGAAGTGCACATATCAATAAGGCTGGTGAAGTGTAGGCTGGAGCTGCTTC	
relA1-rev-P1-rev	CTAACTCCCGTGCAACCGACGCGCGTCGATAACATCCGGCACCATATGAATATCCTCCTTA	
relA6F	ATCCACCAGGTCAATCTTCAC	
relA2633R	AGGATATACCATTGCGCGAC	

### Construction of strains and plasmids

Strain GMF204 (LRT9 Δ*relA*::Km) was obtained by P1 transduction [27] from strain CF1652. Replacement of the Δ*relA*::Km mutation by the *relA* ^+^ allele was performed by co-transducing *relA* ^+^ and *argA*::Tn*10* (38 kDa apart) from strain CF12489 (*argA*::Tn*10 relA* ^+^) into strain GMF204. The transductants were selected for tetracycline resistance and kanamycin sensitivity. The Δ*relA*::Km mutant and the *relA* ^+^ transductant were tested for ppGpp accumulation under amino acid starvation (Figure S1) and also for growth on amino 1,2,4-triazole (AT) plates (not shown).

The Δ*relA*::Cm mutant was constructed by 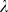 Red recombineering as described [30]. Primers relA1-fw-P1-fw and relA1-rev-P1-rev were used to amplify the CmR gene using plasmid pKD3 as a template. The PCR product was purified from a gel and electrotransformed into strain BW25113 (pKD46). The *relA* deletion was confirmed by PCR and then transduced to strain LRT9. To confirm the Δ*relA* phenotype the resulting strain BS230 (LRT9 Δ*relA*::Cm) was tested for ppGpp accumulation under amino acid starvation and for growth on amino-triazole (not shown). LG01 (LRT9 *lacZ*::Tn*5*) was obtained by P1 transduction from strain MG1655 *lacZ*::Tn*5*.

The BfpA and intimin proteins were tagged with the sequential peptide affinity (SPA) marker to allow the detection of these proteins in immunoblots. The SPA tag is a highly immunogenic 8 kDa peptide composed of a 3XFLAG tag preceded by a calmodulin binding protein epitope, which is recognized with high affinity by the commercially available anti-FLAG M2 antibody. The small size of the SPA tag and its integration next to the carboxy-terminal amino acid makes it unlikely to disturb protein activity [31]. Introduction of the SPA tag into *bfpA* and *eae* was performed essentially as described [31]. Intimin is a 94 kDa protein whose N-terminal is conserved and the C-terminal, which bind to receptors on epithelial cells is highly polymorphic [32]. Thus, to insert the SPA tag in the C terminus of intimin in strain LRT9, we first sequenced the 3′ end of LRT9 *eae* ORF. A 400 bp fragment encompassing the 3′-end of *eae* was amplified by PCR and sequenced. Alignment with the *eae* sequences published in the Genbank database showed that LRT9 carries a class β-1 intimin. To promote DNA recombination in LRT9 the Δ*recBCD*::P*tac-gam-bet-exo* (inducible 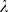-Red system)-*cat* genes were transduced from strain KM32 into LRT9. DNA hybrid primers eae3127(F)-SPA and eae3207(R)-SPA each containing at its 5′-end 40 nt of a sequence immediately upstream or immediately downstream of the *eae* stop codon and the adjacent 20 nt on each primer corresponding to the ends of the SPA-KmR sequence were used to amplify a 1.7 kb DNA fragment with plasmid pJL148 as a template. The PCR product was purified from an agarose gel and electrotransformed into LRT9, giving rise to strain LRT9 *eae*::SPA-Km Δ*recBCD*::P*tac-gam-bet-exo cat*. Elimination of the 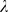-Red genes from LRT9 chromosome was attained by transducing the *argA*::Tn*10* allele from strain CF12489 (the *argA*::Tn*10* marker and the site where the *gam-bet-exo* genes were inserted are adjacent to each other on *E. coli* chromosome). Bacteria were selected for tetracycline resistance and tested for chloramphenicol sensitivity. The resulting strain thus carries the *eae*::SPA-Km fusion but not the 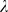-Red genes. LRT9 *eae*::SPA Δ*relA*::Cm was obtained by P1 transduction from strain BS230.

Construction of LRT9 *bfpA*::SPA was essentially as described above for LRT9 *eae*::SPA, except that the hybrid primers used for recombineering (bfpA813(F)-SPA/bfpA893(R)-SPATR were specific for *bfpA*. To confirm that the SPA-tagging of intimin and BfpA was successful, LRT9 *bfpA*::SPA-Km and LRT9 *eae*::SPA-Km were assayed for BfpA and intimin by immunoblotting with a monoclonal anti-Flag antibody, as described below. Bands of the expected sizes, 110 kDa for intimin-SPA and 28 kDa for BfpA-SPA were observed and their levels were higher in DMEM at the exponential phase, and lower in cells grown in LB and in the stationary phase (not shown).

For the construction of plasmid pLG19, the *relA* ORF was amplified by PCR with primers relA6F/relA2633R and cloned in plasmid pTZ57R/T. The resulting plasmid was digested with BamHI/SacI and ligated in the same sites of plasmid pACT3. Plasmid pBS32 was obtained by ligating the GmR gene from pKNOCK-Gm digested with HincII into pLG19 DraI sites.The resulting plasmid pBS32 is Cm^S^ and Gm^R^.

### P1 Transduction

Transduction of chromosomal markers were performed with phage P1 *vir* essentially as described [27]. LRT9 can be transduced with P1 though less efficiently than *E. coli* K-12 strains [33].

### ppGpp assay

For amino acid starvation, exponentially-growing cells were resuspended in T-salts minimal medium [28] supplemented with 0.2% glucose, 0.25 mM ^32^P-KH_2_PO_4_ and grown for 1 h. Amino acid starvation was started by adding 1 mg/ml serine hydroxamate (SH) to the cultures. Samples were collected at several time points following SH addition and immediately mixed with a 0.5 volume of cold formic acid. ppGpp under non-starving conditions was assayed by resuspending an overnight LB culture in DMEM containing only 0.25 mM KH_2_PO_4_ and 100 μCi ^32^P. After 2 hours 0, 1, 10 or 100 μM IPTG were added. ppGpp was extracted and detected as described above. Following overnight incubation at −20°C, the extracts were centrifuged for 5 minutes at 10,000 rpm to precipitate cell debris, and 5 μl were applied to PEI-cellulose TLC-plates. The labeled nucleotides were resolved by one-dimensional TLC using 1.5 M KH_2_PO_4_ as a solvent. The amounts of ppGpp on the chromatograms were estimated by scanning the radioactivity of the spots in a Cyclone Plus storage phosphor system (Perkin Elmer) and calculating the level of ppGpp relative to that of GTP + ppGpp. The densitometric analysis was performed with the help of the software OptiQuant. Alternatively, the TLC plates were exposed to X-ray films and the densitometric analysis of the spots was performed with the software ImageJ [34].

Δ*relA* mutants are sensitive to the histidine analogue Amino-1,2,4-triazole (AT). Plates were prepared as described [29]. LRT9 derivatives were streaked on AT plates and incubated at 37°C for 24–48 h.

### Adherence assay

A suspension containing 10^5^ HEp-2 cells in 1 ml DMEM supplemented with 10% FCS was added to each well of a 24-well tissue plate and grown for 48 h at 37°C with 5% CO2. The medium was removed from the cell monolayer and replaced with 1 ml of fresh DMEM supplemented with 2% FCS and 1% mannose. At this point, 5×10^7^ bacteria previously grown for 18 h in LB medium at 37°C were added to each well. After 3 h of incubation, the cell monolayer was washed six times with phosphate-buffered saline (PBS) to remove the non-adherent bacteria. The monolayer containing the adhered bacteria was treated with 1 ml 0.1% Triton X-100 in PBS for 5 minutes, bacteria were further diluted in PBS, plated onto LB-agar and incubated at 37°C for 24 h. On the next day, colonies were count and the number of colony forming units per ml (CFU/ml) was calculated.

### β-galactosidase assay

Bacteria transformed with plasmid pGM17 were grown overnight in LB medium and diluted 100 times in DMEM. Samples were harvested every hour and assayed for growth (OD600) and β-galactosidase. When plasmid pLG19 was present, increasing concentrations of isopropyl β-D-1-thiogalactopyranoside (IPTG) were added to the cultures as specified in the text. The β-galactosidase assay was performed as described [27]. Briefly, 800 μl of buffer Z (16.1 g/l Na_2_HPO_4_, 5.5 g/l NaH_2_PO_4_, 0.75 g/l KCl, 0.25 g/l MgSO_4_·7H_2_O and 2.7 ml/l β-mercaptoethanol) were added to 200 μl of permeabilized cells and the reaction was started with the addition of 200 μl of a 4 mg/ml ortho-nitrophenyl-galactoside (ONPG) solution. Samples were incubated at 32°C until a yellow color developed and 500 μl of a 1 M Na_2_CO_3_ solution was added to terminate the reaction. The reaction product was read at 420 nm and Miller units were calculated.

### Western-blot analysis

Cells grown overnight in LB were diluted in 10 ml DMEM containing 2% FCS to a final OD_600_ of 0.025 and incubated at 37°C without agitation up to the mid-late exponential phase. Proteins were extracted from 109 bacteria by resuspending in 100 μl of 5× diluted Lane Marker Sample Buffer (Thermo Scientific) and boiling for 5 minutes. 10–20 μl of each sample were resolved by SDS-PAGE and transferred to a nitrocellulose membrane. Immunoblotting was performed with an anti-FLAG M2 primary antibody (Sigma) followed by a peroxidase conjugate of anti-mouse imunnoglobulin G as secondary antibody. Protein bands were detected using the SuperSignal West Pico Chemiluminescent system (Pierce), as recommended by the manufacturer.

### Sequencing of LRT9 *eae* 3′-end

For the sequencing of the highly polymorphic 3′-end of *eae* a DNA fragment of 369 bp was amplified by PCR with primers eae3036F/eae3536R using E2348/69 or LRT9 colonies as templates. The PCR products were purified directly with Wizard DNA Preps DNA purification system (Promega). DNA was sequenced by the Sanger method in an automatic sequencer type ABI Prism 3100 Genetic Analyzer (Applied Biosystems/Hitachi, Warrington, UK).

## Results

### Effect of Δ*relA* on adherence

**Figure 1:**
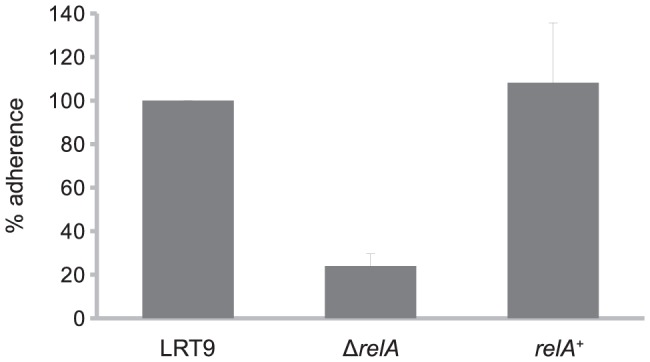
Effect of *relA* on the adherence of EPEC to epithelial cells. 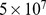
 bacteria grown overnight in LB broth were transferred to HEp-2 cells monolayers in DMEM supplemented with 2% FBS and incubated for 3 h. The cell wells were washed and the bacteria were released, diluted and plated on LB agar for CFU counting. To the wild-type strain was attributed the value of 100%. Each column represents the mean standard error of the mean (S.E.M.) of three independent experiments.

To study the effect of *relA* on EPEC adherence, the Δ*relA*::Km allele was transferred to the EPEC strain LRT9. The LRT9 Δ*relA*::Km mutant did not accumulate ppGpp under amino acid starvation (Fig. S1) and was sensitive to amino-triazole (not shown), confirming the *relA* deficiency. To test the effect of Δ*relA* on EPEC adherence to epithelial cells, a standard *in vitro* adherence assay was conducted [33], [35]. Bacteria grown overnight in LB were transferred to 24-well plates containing HEp-2 cells monolayers in DME medium and incubated for 3 hours to allow their adherence to the HEp-2 cells. The level of bacterial adherence was determined by counting the number of viable bacterial cells adhered to the HEp-2 cells monolayer at the end of the incubation period (Figure 1). Adherence of the Δ*relA* mutant was 75% lower than that of the *relA* ^+^ parent (LRT9). Normal levels of adherence were restored when the *relA* ^+^ allele was transduced back into the Δ*relA*::Km mutant (strain *relA* ^+^ *argA*::Tn*10*). The inhibition in adherence caused by the *relA* deletion cannot be attributed to differences in growth rate, as the wild-type and the Δ*relA* mutant displayed very similar growth curves under the conditions employed in this assay (see below).

### Effect of Δ*relA* on the expression of the *perABC* operon

**Figure 2:**
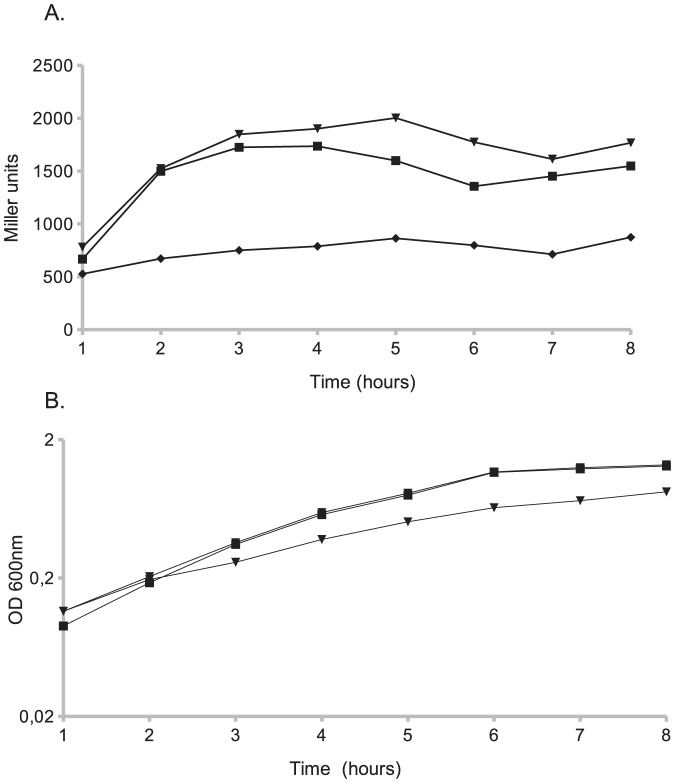
Effect of *relA* on P*per* expression. Transformants carrying plasmid pGM17 (P*per*-*lacZ*) were grown overnight in LB, resuspended in DMEM and grown for 8 hours without agitation. Samples were taken every hour and assayed for (A) β- galatosidase activity and (B) growth (OD_600_). (▪) wild-type (LRT9); (⧫) Δ*relA* (GMF204); (▾) pLG19 (*relA* ^+^) in GMF204. Each point represents the average of two independent experiments.

In typical EPEC strains, both primary and intimate adherence are regulated by the products of the *perABC* operon. Transcription of the *bfp* and *perABC* operons [6], [36] is dependent on PerA, while PerC is required for the synthesis of the master regulator Ler, which in turn activates the transcription of the LEE genes, including *eae* that encodes intimin [12], [37]. To test whether the Δ*relA* mutation affects the expression of the *perABC* promoter plasmid pGM17 which harbors a P*per*-*lacZ* fusion was transformed into LRT9 and into LRT9 Δ*relA*::Km. The same growth conditions used in a standard adherence assay (Fig. 1) were employed here. Bacteria grown overnight in LB, were diluted and further grown in DMEM without agitation for 8 h. Samples were harvested every hour and assayed for β-galactosidase activity (Fig. 2A). Expression of the *per* promoter (P*per*-*lacZ*) in LRT9 increased three-fold as cells grew through the exponential phase reaching a peak at 4 h. The activity of P*per*-*lacZ* in the Δ*relA* mutant was in average twice as lower as in the wild-type strain. For some unknown reason the *relA* ^+^ *argA*::Tn*10* transductant failed to fully restore the P*per* original activity (not shown). To overcome this difficulty, the Δ*relA* mutant was transformed with plasmid pLG19 (*relA* ^+^ cloned in a low-copy vector under P*tac*). pLG19 not only complemented the mutation, but even slightly increased the expression of P*per* when compared to the wild-type strain. This surplus activity is likely to be caused by the leakiness of P*tac*, which is active even in the absence of IPTG increasing thus the cellular level of ppGpp (see below). Figure 2B shows the growth curves of the bacterial strains. The wild-type strain and the Δ*relA* mutant displayed similar growth curves throughout the experiment. At the exponential phase (1–6 h) the growth rate was μ  =  0.51 h^−1^ for LRT9, μ =  0.46 h^−1^ for the Δ*relA* mutant and μ =  0.34 h^−1^ for the pLG19 transformant. The slower growth of the latter is in part due to its slightly higher ppGpp level in the absence of IPTG (0.34 units of ppGpp in the transformant against 0.28 units of ppGpp in the non-transformed strain).

**Figure 3:**
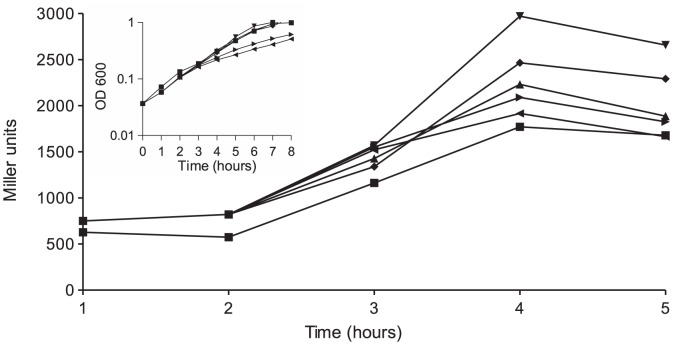
Effect of ppGpp overproduction on P*per*. LG01 (LRT9 *lacZ*::Tn*5*) carrying pGM17 (P*per*-*lacZ*) and LG01 carrying pGM17 and pLG19 (P*tac*-*relA* ^+^) were grown and sampled as in the legend to Figure 2. After 2 h of growth the bacteria were divided into five sub-cultures and (⧫) 0, (▾) 0.001, (▴) 0.01, (▸) 0.1 or (◂) 1.0 mM IPTG were added; (▪) represents the non-transformed wild-type strain. The insert shows the growth curves from 0 to 8 h. Each point represents the average of two independent experiments.

To further examine the effect of ppGpp on the expression of the *perABC* operon, plasmid pLG19 was transformed into LRT9 *lacZ*::Tn*5* and into LRT9 Δ*relA*::Km *lacZ*::Tn*5* carrying pGM17 (P*per*-*lacZ*). β-galactosidase activity was followed as above, except that IPTG (0.001, 0.01, 0.1 or 1.0 mM) was added to the cultures (Figure 3). Similarly to what was observed in Figure 2A the mere presence of pLG19 increased P*per-lacZ* expression when compared to the non-transformed wild-type strain. Addition of 1 μM IPTG further boosted β-galactosidase activity, suggesting that a small increase in ppGpp positively affects *perABC* transcription. Higher concentrations of IPTG did not further stimulate P*per*-*lacZ* activity because high levels of ppGpp cause growth arrest and general protein inhibition, limiting the beneficial effect of ppGpp on gene expression [23], [38], [39]. Indeed, the insert in Figure 3 shows that addition of 0.1 and 1.0 mM IPTG caused a strong inhibition in growth rate. Figure S2 shows that LRT9 carrying pLG19 responds to increasing concentrations of IPTG in the medium by accumulating more ppGpp.

### Effect of *relA* on EPEC adhesins

**Figure 4:**
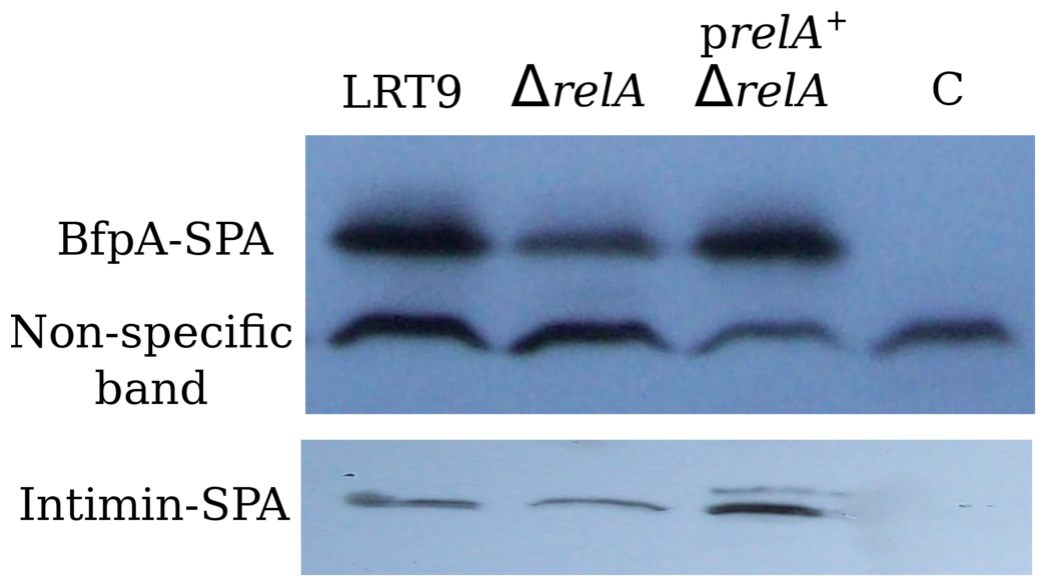
Effect of *relA* on the synthesis of BfpA and intimin. Bacteria grown overnight in LB broth were resuspended in DMEM and grown up to the mid-exponential phase. At this point the bacteria were harvested and immunoblotted with anti-SPA (FLAG 3X) antibodies. LRT9, wild-type strain; Δ*relA*, LRT9 Δ*relA*::Cm; p*relA* ^+^ Δ*relA*, pLG19 transformed in Δ*relA*::Cm; C, control strain - LRT9 not carrying a *bfpA*::SPA or *eae*::SPA fusion. The picture is representative of two independent experiments.

The type IV pilus BFP and the outer membrane protein intimin are the main adhesins of EPEC, both are positively regulated by the products of the *perABC* operon. Once ppGpp was shown to stimulate adherence and the transcription of the *perABC* operon, the next logical step was to test whether the synthesis of the EPEC adhesins was also affected by Δ*relA*. A Sequential Peptide Affinity (SPA) tag [31] was inserted into the C-terminus of BfpA and intimin to detect the proteins by immunoblotting. The SPA DNA sequence was introduced by 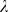-*red* recombineering into the last codon of *bfpA* and *eae* ORFs (see Methods). Because the SPA-tag is linked to a kanamycin resistance gene, a new Δ*relA*::CmR mutant was constructed in a K-12 background and transduced into LRT9. The new LRT9 Δ*relA*::Cm mutant was tested for growth on amino-triazole and for ppGpp accumulation under amino acid limitation (data not shown). Likewise, the Cm^R^ marker in pLG19 (p*relA* ^+^) was replaced by Gm^R^ to allow complementation *in trans* of the Δ*relA*::Cm^R^ mutation. To assess the protein levels of BfpA and intimin, bacteria were grown under the same conditions used in the adherence assay, i.e., overnight growth in LB broth, followed by growth in DMEM without agitation. Proteins were extracted at the mid-late exponential phase, resolved by SDS-PAGE and immunoblotted with the anti-Flag monoclonal antibodies. Figure 4 shows that the bands corresponding to BfpA-SPA and intimin-SPA were considerably less intense in the Δ*relA* mutant than in the wild-type strain. Introduction of pBS32 (p*relA* ^+^) not only complemented the mutation, but further increased the level of BfpA and intimin (compare the band intensities of the pBS32 transformant to that of the non-specific band). This indicates once more that a small increase in ppGpp has a positive effect on the synthesis of proteins associated with EPEC adherence. It can be concluded that *relA*/ppGpp play a positive role in the synthesis of the BFP fimbria and intimin and consequently, on EPEC adherence.

## Discussion

The alarmone ppGpp controls many and diverse aspects of bacterial metabolism. Chiefly, ppGpp plays a pivotal role in maintaining the equilibrium between the intracellular amino acid pool and the rate of protein synthesis. This function is mediated by the ppGpp-synthetase RelA, which captures the signal from uncharged tRNAs at the ribosome A site and initiates the synthesis of ppGpp [16].

The present study provides evidence that the adherence of EPEC to epithelial cells and the expression of genes associated with adherence are positively regulated by ppGpp. This conclusion is supported by the following findings: deletion of *relA* significantly inhibited the adherence of EPEC to epithelial cells *in vitro*, reduced the protein levels of the adhesins BFP and intimin and inhibited the expression of the *perABC* operon. Furthermore, mild overproduction of ppGpp had a beneficial effect on the expression of *bfp*, *eae* and *perABC*.

In typical EPEC, transcription of the LEE and of the *bfp* operon depends, respectively, on PerA [6], [36] and PerC [12]. Sequence analysis of the *perABC* promoter in the LRT9 strain revealed an AT-rich discriminator sequence (-10 AAAATCAATAG +1), compatible with promoters activated by ppGpp [40], [41]. In AT-rich promoters ppGpp destabilizes the RNA polymerase (RNAP)-promoter complex allowing RNAP to escape and to carry out the elongation of the RNA chain [41]. It is thus been suggested that ppGpp stimulates EPEC adherence by enhancing the transcription of *perABC*, which in turn upregulates BFP and intimin.

Another gene involved in ppGpp metabolism, *spoT*, encodes a bifunctional enzyme with a weak synthetase and a strong hydrolase activity [19]. *spoT* cannot be deleted from *E. coli* chromosome unless in a *relA* background because ppGpp over-accumulation would result in bacterial growth arrest. We managed to construct a ppGpp^0^ mutant (Δ*relA* Δ*spoT*) in LRT9. The inhibitory effect of the double mutant on adherence and on P*per-lacZ* activity was, if anything, stronger than that of the *relA* single deletion (data not shown). However, the growth rate of the double mutant in DMEM was severely impaired, probably owe to the multiple auxotrophies caused by the complete absence of ppGpp in this strain [19]. It was therefore unclear to what extent the effect of the double deletion was caused by the lack of ppGpp or due to a deleterious effect on growth.

The assay of EPEC adherence to epithelial cells is usually performed by first growing the bacteria overnight in the nutrient-rich LB medium, followed by their transfer to HEp-2 cells monolayers seeded in DMEM [35]. The passage from an amino acid rich medium (LB) to a relatively poor medium such as DMEM mimics the pathway of EPEC through the digestive system, alternating portions of high nutrient content in the jejunum and the lower part of the small intestine, where nutrients are less abundant. Under these conditions RelA is likely to be activated, and a temporary increase in ppGpp ensues. Accordingly, EPEC adherence genes are poorly expressed in rich media [4], [42] when ppGpp concentration is low and are activated upon transferring to DMEM.

The bacterial growth rates observed in the present study (0.4–0.5 h^−1^) are similar to the one reported for *E. coli* in the host intestine [43], where *in vivo* adherence occurs. ppGpp is intimately associated with growth control and as shown here, it helps modulating the synthesis of EPEC adhesins. However, ppGpp level should not be as high as to cause complete growth arrest. We were able to reproduce the optimal concentration of ppGpp that results in the highest level of expression of the adherence-related genes by cloning *relA* in a low-copy plasmid under the control of P*tac* and inducing the promoter with low concentrations of IPTG (Figs 3B and S2).

It has previously been shown that ppGpp enhances the expression of the LEE in enterohemorrhagic *E. coli* (EHEC) [44]. However, there are some important differences between EPEC and EHEC regarding the expression of the adhesin genes. First, even though both pathotypes share the LEE pathogenicity island, the EAF plasmid is not present in EHEC strains and the LEE genes are thus not regulated by PerC. Second, transcription of the LEE genes in EHEC begins at the mid-exponential phase and peaks at the late exponential/early stationary phase [45]. In contrast, in EPEC the LEE as well as the *perABC* and *bfp* operons are maximally activated at the mid-exponential-phase (Fig. 3 and [4]). Third, expression of the LEE genes as well as the adherence capacity of EHEC are higher in LB medium supplemented with bicarbonate [44], while EPEC neither adhere nor properly expresses the genes associated with adherence when grown in LB supplemented or not with bicarbonate ([4], [11], [42], [46] and data not shown). Even though the environmental conditions required for the synthesis of EPEC and EHEC adherence factors are not identical, ppGpp positively affects the adherence of both lineages. This suggests that regulation by ppGpp is conserved regardless the specific mechanisms of control of adherence employed by the different diarheogenic bacteria.

ppGpp is associated with bacterial virulence in several species. In most cases ppGpp plays a positive role and is required to fully induce the virulence genes. For instance in all *Proteobacteria* hitherto analyzed, such as *E. coli* (EHEC and UPEC), *Salmonella enterica*, *Yersinia pestis*, *Pseudomonas aeruginosa Francisella tularensis* and *Bordetella pertusis* a positive role for ppGpp was found [24], [44], [47]–[53]. This reinforces the notion that upregulation of bacterial virulence by ppGpp is an ancient evolutionary phenomenon.

## Conclusions

The present study shows that the ppGpp synthetase RelA is required for the maximal adherence of EPEC to epithelial cells and for the synthesis of the adhesins BFP and intimin. ppGpp positively affects the transcription of the *perABC* operon that encodes the main adherence regulators PerA and PerC. PerA positively controls the transcription of the *bfp* operon, while PerC activates the transcription of LEE1, whose product Ler, activates LEE5 (*cesT-tir-eae*) that encodes intimin. Therefore, by enhancing *perABC* ppGpp will ultimately upregulate adherence. EPEC can thus be added to the list of pathogenic bacteria whose virulence is enhanced by ppGpp.

## Supporting Information

Figure S1 **ppGpp accumulation in amino acid starved bacteria.** Bacteria were treated with serine hydroxamate for 30 minutes. Samples were withdrawn at 15 and 30 minutes and ppGpp was resolved by TLC.
(EPS)

Figure S2 **Accumulation of ppGpp caused by overproduction of** ***relA*** **.** Exponentially growing bacteria (LRT9 carrying plasmid pLG19) in DMEM in the presence of 100 _Ci 32P were treated with IPTG for 3 h. Samples were harvested each hour and assayed for ppGpp.
(EPS)

File S1 **Supporting information.**
(PDF)
