# Supplementary material for: relA Enhances the Adherence of Enteropathogenic Escherichia coli
Source: PLoS One. 2014 Mar 18;9(3):e91703. doi: 10.1371/journal.pone.0091703 (PMC3958371; doi:10.1371/journal.pone.0091703)
Supplement: File S1 — Supporting information. (PDF) [file pone.0091703.s003.pdf]

SUPPORTING INFORMATION

relA enhances the adherence of enteropathogenic *Escherichia coli*

Beny Spira, Gerson Moura Ferreira, Luiz Gustavo de Almeida

Departamento de Microbiologia, Instituto de Ciências Biomédicas, Universidade de São Paulo, São Paulo-SP, Brazil

ppGpp synthesis in LRT9 derivatives

To confirm the genotypes of the Δ*relA* mutant and of the backcrossed transductant *relA*$^+$ *argA*::Tn*10*, ppGpp accumulation under amino acid starvation was tested. Bacteria were grown in TGP minimal medium (Tris-buffered minimal medium supplemented with 0.2% glucose, 0.25 mM KH_2PO_4, 100 μg/ml arginine and 100 μCi ^{32}P-H_3PO_4). At time 0, 20 μl samples were withdrawn and mixed with 10 μl cold formic acid. 1 mg/ml serine hydroxamate was then added to induce amino acid starvation and samples were withdrawn at 15 and 30 minutes. ppGpp was resolved on a TLC plate with 1.5 M KH_2PO_4 as the mobile phase. The TLC plate was then dried and exposed to an X-ray film. Figure S1 shows that, as expected, both pppGpp and ppGpp accumulated in the wild-type (LRT9) and in the *relA*$^+$ *argA*::Tn*10* strain. The Δ*relA* mutant displayed a typical relaxed response with a reduction in ppGpp level. These data confirmed the genotypes of the strains used in the present study.

The level of ppGpp in LRT9 transformed with pLG19 (p*relA*$^+$) was assayed by growing bacteria under the same conditions as above , except that instead of SH, at time 2 h (in Fig. 3 - insert), 0, 1, 10 or 100 μM IPTG were added. ppGpp was extracted and detected as described above. Figure S2 shows that there was a steady increase in ppGpp in all cultures accompanied by a decrease in growth rate (Fig. 3 insert). As expected, higher concentrations of IPTG resulted in higher ppGpp levels. The increase in ppGpp throughout the incubation period even in the absence of IPTG is probably related to the deterioration in growth conditions, such as higher cell density and oxygen shortage (the cultures were not under agitation).

Supporting information legends

Figure S1. ppGpp accumulation in amino acid starved bacteria. Bacteria were treated with serine hydroxamate for 30 minutes. Samples were withdrawn at 15 and 30 minutes and ppGpp was resolved by TLC.

Figure S2. Accumulation of ppGpp caused by overproduction of relA. Exponentially growing bacteria (LRT9 carrying plasmid pLG19) in DMEM in the presence of 100 μCi ^{32}P were treated with IPTG for 3 h. Samples were harvested each hour and assayed for ppGpp.
